# New Appliances and Things Medical

**Published:** 1908-12-19

**Authors:** 


### NEW APPLIANCES AND THINGS MEDICAL.

[We shall be glad to receive at our Office, 28 & 29 Southampton Street, Strand, London, W.C., from the manufacturers, specimens of all new preparations and appliances which may be brought out from time to time.]

"No. 4711" Eau de Cologne, which has been distilled for very many years by the firm of Ferdinand Mülhens, of No. 4711 Rue de la Cloche, Cologne, is already well known in this country. Medical men can be quite satisfied that, in recommending this brand of Eau de Cologne to their patients, they are making no mistake. Only the best varieties retain their freshness and fragrance after being several hours on the pocket handkerchief. "No. 4711" is a delicate and refreshing perfume, and has excellent lasting properties. Eau de Cologne is almost the only scent which has medicinal uses, and which can be tolerated by the fastidious nostrils of an invalid, and it is important that none but the best brands be allowed to enter the sick-room. The variety under notice bears a tasteful and characteristic label of blue and gold, which is easily recognised, and those who purchase it may be sure that they are obtaining a really good Eau de Cologne. The agent for this country is R. J. Reuter, 5-7 Denman Street, London, W.

### A FORMALIN CONE FOR FUMIGATION.

"Formozone" is a new and effective preparation of formaldehyde for fumigating sick-rooms, hospital wards, public halls, offices, cellars, school-rooms, railway carriages, etc. The formaldehyde is incorporated in a black carbonaceous substance which is pressed into the shape of a cone. A copper wire is inserted in the base of the cone, which by this means can be suspended, apex downwards, over a pail. The application of a lighted match to the apex is all that is now required for the slow combustion of the material. When the Formozone is ignited Formaldehyde gas is slowly generated in an active form, and each cone is stated to contain sufficient solidified Formalin to disinfect a room of 1,000 cubic feet. We have examined these cones, and are satisfied they can easily be used according to the directions given, and that, when ignited and placed in the middle of a room, they evolve a considerable quantity of active formalin vapour, which, as is well known, is nowadays regarded by public health authorities as the most reliable and efficient fumigating agent for house disinfection. The simplicity of this method should recommend it to medical practitioners, school managers, householders, and others, at times when infectious diseases are prevalent. It is also well suited for cleansing and deodorising the foul air of public vehicles and lavatories. Each cone is packed in sawdust in a tin cylinder, and is sold at the price of 6d. The manufacturers are Messrs. Charles Zimmerman and Company, 9 and 10 St. Mary-at-Hill, London, E.C.

